# Next-generation neuropeptide Y receptor small-molecule agonists inhibit mosquito-biting behavior

**DOI:** 10.1186/s13071-024-06347-w

**Published:** 2024-06-28

**Authors:** Emely V. Zeledon, Leigh A. Baxt, Tanweer A. Khan, Mayako Michino, Michael Miller, David J. Huggins, Caroline S. Jiang, Leslie B. Vosshall, Laura B. Duvall

**Affiliations:** 1 Laboratory of Neurogenetics and Behavior, The Rockefeller University, New York, NY 10065 USA; 2 Howard Hughes Medical Institute, New York, NY 10065 USA; 3 Sanders Tri-Institutional Therapeutics Discovery Institute, New York, NY 10065 USA; 4 Present Address: Atai Life Sciences, New York, NY 10012 USA; 5 Department of Physiology and Biophysics, Weill Cornell Medicine, New York, NY 10065 USA; 6 Center for Clinical and Translational Science, The Rockefeller University, New York, NY 10065 USA; 7 Kavli Neural Systems Institute, New York, NY 10065 USA; 8 Department of Biological Sciences, Columbia University, New York, NY 10027 USA

**Keywords:** Mosquito, *Aedes aegypti*, Neuropeptide Y, Host-seeking, Small-molecule agonist, Structure-guided design

## Abstract

**Background:**

Female *Aedes aegypti* mosquitoes can spread disease-causing pathogens when they bite humans to obtain blood nutrients required for egg production. Following a complete blood meal, host-seeking is suppressed until eggs are laid. Neuropeptide Y-like receptor 7 (NPYLR7) plays a role in endogenous host-seeking suppression and previous work identified small-molecule NPYLR7 agonists that inhibit host-seeking and blood-feeding when fed to mosquitoes at high micromolar doses.

**Methods:**

Using structure–activity relationship analysis and structure-guided design we synthesized 128 compounds with similarity to known NPYLR7 agonists.

**Results:**

Although in vitro potency (EC_50_) was not strictly predictive of in vivo effect, we identified three compounds that reduced blood-feeding from a live host when fed to mosquitoes at a dose of 1 μM—a 100-fold improvement over the original reference compound.

**Conclusions:**

Exogenous activation of NPYLR7 represents an innovative vector control strategy to block mosquito biting behavior and prevent mosquito–human host interactions that lead to pathogen transmission.

**Graphical Abstract:**

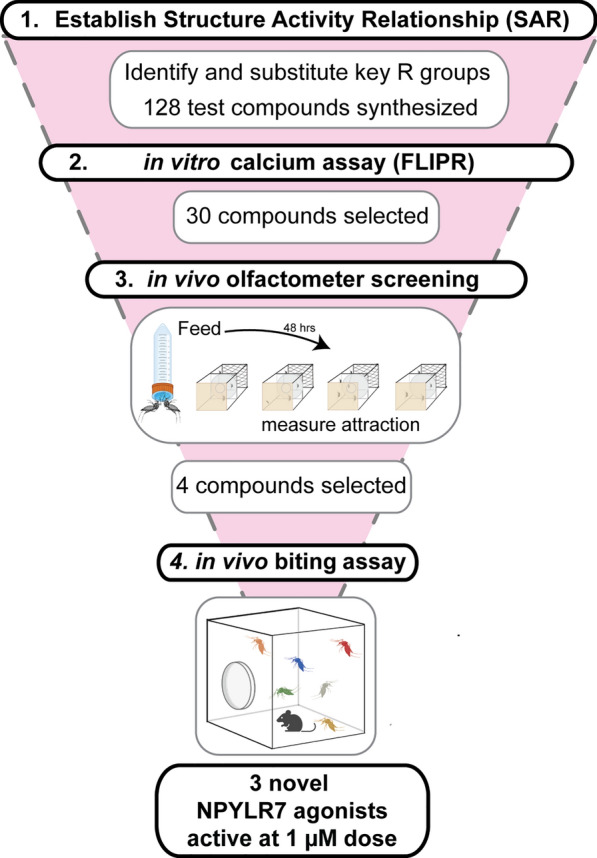

**Supplementary Information:**

The online version contains supplementary material available at 10.1186/s13071-024-06347-w.

## Background

Female *Aedes aegypti* mosquitoes are innately attracted to find and bite human hosts to obtain blood protein required for egg development. However, host-seeking behavior is regulated by the female’s internal state and is naturally suppressed after a full meal of blood during egg development [1]. Blood nutrients are required for sustained suppression; although female mosquitoes will engorge on non-nutritive saline meals that cannot support egg development, these females return to high levels of host-seeking when abdominal distensions wear off roughly 24 h later [2–4]. Previous work implicates abdominal mechanosensors in mediating short-term suppression and neuropeptide pathways in mediating sustained, days-long, suppression [2, 3, 5–10]. Neuropeptide Y-related pathways regulate hunger and satiety in many organisms [11–15], and we recently identified *Ae. aegypti* neuropeptide Y-like receptor 7 (NPYLR7) as a key regulator of host-seeking after a blood meal [2]. After blood-feeding, NPYLR7 activation acts as a satiety signal and suppresses attraction to hosts. Pharmacological activation of NPYLR7 inhibits biting and blood-feeding even in the absence of blood nutrients. Conversely, female mosquitoes with genetically or pharmacologically disrupted NPYLR7 signaling continue to host-seek inappropriately after a blood meal. Exogenous activation of feeding-related neuropeptide receptors in mosquitoes represents a novel approach for blocking their attraction to humans by exploiting the pathways that naturally suppress the drive to bite.

Here, we used structure–activity relationship analysis and structure-guided design to identify novel small-molecule NPYLR7 agonists with improved in vitro and in vivo potency relative to compounds identified in a previous high-throughput small-molecule screen of 265,211 compounds [2]. We synthesized 128 new compounds and characterized their in vitro potency using a human embryonic kidney (HEK) cell-based assay to evaluate NPYLR7 activation. To identify those with in vivo activity, we tested 30 compounds in a host-seeking screening assay and subsequently identified three compounds that significantly reduced blood-feeding from a live host when delivered to mosquitoes at a dose of 1 μM—100-fold lower than that used in our original report [2].

Although three out of 30 compounds tested in vivo reduced host-seeking behavior, in vitro potency was not highly predictive of in vivo efficacy. This disconnect highlights the need for intermediate assays to span the gap between cell-based and behavioral assays in mosquitoes. This work is important because it identifies new highly potent compounds that block mosquito blood-feeding by targeting a neuropeptide receptor that regulates mosquito attraction to humans through a conserved satiety pathway.

## Methods

### Molecular modeling for the NPYLR7 agonist series

The docking model for the NPYLR7 agonist series was generated based on a homology model of *Ae. aegypti* NPYLR7 and validated with rigorous FEP+ binding energy calculations [16]. The homology model of *Ae. aegypti* NPYLR7 was obtained from the GPCR-I-Tasser homology modeling server (https://zhanggroup.org/GPCR-I-TASSER/) [17]. To predict the binding mode of reference compound TDI-012631, the compound was docked to the orthosteric binding site of the NPYLR7 homology model using Glide SP, then the receptor–ligand complex was refined in a POPC lipid bilayer environment using Desmond molecular dynamics (MD) simulation for 120 ns at a constant temperature of 300 K in NPγT ensemble. The protein structure was prepared using the Protein Preparation Wizard in Maestro with default settings. The ligand structure was prepared using LigPrep in Maestro. The protonated form of the ligand was selected, according to the predicted pKa of the guanidine moiety (pKa = 10.21 in Jaguar). The MD simulations were carried out using the OPLS3e force field [18]. The final snapshot at 120 ns was minimized and then subjected to an absolute FEP+ calculation, [19] which showed favorable binding energy (ΔG = −18.02 ± 0.24 kcal/mol). To further validate the docking model, relative FEP+ calculations were performed on a validation set of 13 compounds in the series having half maximal effective concentration (EC_50_) values ranging across three log units. The validation showed good agreement between the experimental and FEP+ predicted potencies (mean unsigned error [MUE] = 1.29 kcal/mol; *R*^2^ = 0.57). The perturbation maps were automatically generated using the Mapper tool. Force Field Builder was employed to generate custom torsional parameters for ligand torsions that were not included in the default force field. FEP+ calculations were run for the default 5 ns. The Schrödinger Suite was used for protein and ligand preparations, docking, MD, and FEP+ calculations (release 2020-4, Maestro, Schrödinger LLC, New York, NY, USA).

### Synthetic methods for analog preparation

Unless otherwise noted, the following pertain to the synthetic methods: all reactions are magnetically stirred; typical solvents (ethyl acetate, hexanes, dichloromethane, and methanol) are Fisher Optima grade; “concentrated to dryness” or “removal of the solvent” means evaporating the solvent from a solution or mixture using a rotary evaporator; flash chromatography is carried out on an Isco, Analogix, or Biotage automated chromatography system using a commercially available cartridge as the column. Columns are usually filled with silica gel as the stationary phase; preparative high-performance liquid chromatography (HPLC; or prep-HPLC) is carried out with commercial columns in a reverse phase manner (the stationary phase is hydrophobic). Typical solvent mixtures include A (water) and B (organic, i.e., acetonitrile, methanol, etc.). Additives can also be used in the solvent mixture such as HCl, NH_4_HCO_3_, and formic acid. Details for individual synthesis reactions are provided in Supplemental Methods.

### In vitro assay

The in vitro screening assay was adapted from [2], and carried out at HD Biosciences (HDB, Shanghai) as follows: HEK293T (Thermo Fisher Scientific) cells were grown in Dulbecco’s modified Eagle medium (DMEM) (high glucose, with glutamine), 10% fetal bovine serum, 1% penicillin–streptomycin (Pen/Strep), seeded in a 75 cm^2^ flask, and incubated at 37 °C and 5% CO_2_. Cells were transiently transfected with 1 μg of each plasmid expressing GCaMP6s (Addgene #277314.1040753) (Chen et al., 2013), mouse Gqα15 (Addgene #40753) (Offermanns and Simon, 1995) and *Ae. aegypti* NPYLR7 (Addgene #52392) (Duvall et al. 2019) using Lipofectamine 2000 (Invitrogen) in 4 ml of Opti-MEM (Invitrogen). This mixture was added to a plate after 20 min and incubated for 6–8 h. Cells were then trypsinized, resuspended in phenol-free media, and plated in a 384-well plate (Greiner Bio-One) at a density of 20,000 cells per 40 μl, and incubated overnight (37 °C, 5% CO_2_). Cells were imaged directly in phenol-free media. Plates were loaded into a Molecular Devices fluorescence imaging plate reader (FLIPR) with an excitation wavelength of 470–495 nm and an emitted wavelength of 515–575 nm. Plates were imaged every second for 5 min. After 30 s of baseline fluorescence recording, 10 μl of the test compound in reading buffer [Hank’s Balanced Salt Solution (Gibco) + 20 mM HEPES (Sigma-Aldrich)] was added. Concentrations tested ranged from 0 to 100 μM. Data were collected in raw fluorescence units (RFU).

### EC_50_ calculations

Each test plate included replicates of 10 μM FMRFa3 (H‐Ala‐Gly‐Gln‐Gly‐Phe‐Met‐Arg‐Phe‐NH2), an *Ae. aegypti* neuropeptide agonist of NPYLR7 used to calculate the hundred percent effect (HPE). The zero percent effect (ZPE) control was calculated as a response to buffer alone. The Z′ factor was calculated for each plate by ${Z}^{\prime}=1-\frac{{3\sigma }_{HPE}+{3\sigma }_{ZPE} }{|{\mu }_{HPE- }{\mu }_{ZPE}|}$. Plates with Z′ < 0.5 were excluded from analysis. Raw fluorescence units (RFU) were converted to percent effect relative to the average of the ZPE and HPE. Percent effect = (sample luminescence − average ZPE)/(average HPE − average ZPE)×100. Doses ranged from 0 to 100 μM, with the highest dose of 100 μM excluded in replicates in which this dose showed aberrant responses beyond the plateau (defined as 2 points with < 15% change). Each replicate was plotted as percent effect versus log[concentration], and replicates with maximal responses between 20 and 250% were used for EC_50_ calculations. Relative EC_50_ was calculated using an [Agonist] versus response–variable slope four-parameter model where $Y=\text{Bottom} +\frac{ ({X}^{\text{Hillslope}})*(Top-\text{Bottom})}{({X}^{\text{HillSlope}}+ {EC50}^{\text{HillSlope}})}$. For compounds with multiple replicates, the relative EC_50_ of each replicate was averaged.

### Mosquito rearing and maintenance

*Aedes aegypti* (Orlando strain) were reared and maintained in a STERIS environmental room under the following conditions: 26–28 °C, 80% humidity. Light cycles consisted of 14:10 h (L:D). Eggs were hatched in hatching broth: one fish food tablet (TetraMin Tropical Tablets, PetMountain) crushed using a mortar and pestle brought to a volume of 850 ml diH_2_O and autoclaved for sterility). During larval stages (days 2–5), larvae were fed two tablets of fish food per day. Pupae were collected and allowed to eclose in 91 cm × 61 cm × 61 cm BugDorm cages (BioQuip Products). Adults were provided with cotton dental wicks (Richmond Dental) inserted into Boston clear round 60 ml glass bottles (Thermo Fisher) filled with 10% sucrose (w/v). Adults were co-housed with siblings and allowed to mate freely for 7 days post-eclosion. All behavioral experiments were performed using 14–21-day-old females as in [2]. Males were removed prior to all behavioral experiments.

### Glytube feeding

Groups of 60–150 females were placed in bucket cages with a diameter of 21.6 cm and height of 16.5 cm (VWR) and fasted with access to water for 24 h prior to feeding. Meals were delivered to females via Glytube membrane feeders as described previously [20]. Meals consisted of 1.5 ml sheep blood (HemoStat Laboratories), saline (400 mM NaHCO_3_ + dih_2_O), or saline and test compound. Lyophilized compounds were stored at room temperature until use. High-concentration stocks (30 mM in 100% dimethyl sulfoxide [DMSO]) (Sigma-Aldrich) were stored at −20 °C and diluted in saline immediately prior to feeding. Once prepared, meals and glycerol heating elements were warmed in a 42 °C water bath for 15 min to provide warmth to attract mosquitos to feed. ATP (1 mM final concentration) and test compounds (1 μM final concentration) were added prior to placing the meal on top of the mesh on the cage containing female mosquitos. Females were allowed 15–20 min to feed to repletion. Abdominal engorgement was scored by eye and confirmed by weighing females to ensure full feeding (Data S1). Females that did not feed to repletion were discarded. Females were returned to their original cage with a diH_2_O wick for 48 h prior to behavioral testing. The lethality of each compound was measured by counting the number of dead females in each cage 24 h post-meal. Any compounds that resulted in a death rate > 50% were scored as “high lethality” and excluded from behavioral testing.

### Behavior

#### Host-seeking screening assay (miniport olfactometer)

Miniport olfactometers were fabricated in-house as described previously [2]. Information regarding design, construction, and use can be found at https://github.com/VosshallLab/Miniport-Construction. Miniport canisters consisted of a 6″ × 3″ × 3″ acrylic tube with a mesh screen to allow for air flow. Groups of 10–20 females were loaded into canisters 24 h post-meal. Females were left in canisters overnight to acclimate with two cotton balls soaked in diH_2_0 to prevent desiccation. Behavior trials began 48 h post-meal. Canisters were randomly assigned to attraction traps 1–4 and given 5 min to acclimate. The stimulus end of the trap was connected to a flowmeter that supplied 5% CO_2_ at a rate of 30 ml/min. Thirty seconds of CO_2_ flow was supplied to activate females prior to opening the sliding door. Once opened, the sliding door gave females access to the attraction trap baited with a human-scented nylon stocking previously worn by the same experimenter for 8–10 h to collect body odor and stored in a plastic bag at −20 °C until use. After 5 min, the sliding door was closed and attraction was scored as mosquitoes in the attraction trap/total number of mosquitoes. For each experiment data were normalized to the matched average saline attraction from the same experiment (% attracted/average saline attraction). Any dead mosquitoes were excluded from the analysis. Non-fed females and blood-fed females served as environmental control groups while the saline-fed females served as the vehicle control each day. If non-fed or saline-fed females were less than 50% attracted or if blood-fed females were more than 20% attracted behavioral trials were halted and all data from that experimental day were discarded.

#### Biting assay (mouse-in-cage)

This assay was modified from [2]. Females were fed and scored as described above. Forty-eight hours post-meal, females were anesthetized in a 4 °C cold room and aspirated into a petri dish (Thermo Fisher Biosciences) with a randomly assigned color powder (Slice of the Moon; Chameleon Colors) to mark their treatment group. Females were then individually removed from the color powder, placed inside a cage measuring 91 cm × 61 cm × 61 cm (BugDorm), and allowed 2–4 h to recover. Each experiment consisted of a cage with 12–20 females in each treatment group including environmental (non-fed and blood-fed) and vehicle (saline-fed) control groups. The experiment began once an anesthetized mouse was introduced to the center of the cage, and females were allowed 15 min to blood-feed. The mouse was then removed, and the cage was placed at 4 °C to anesthetize females for collection. Females were aspirated into a large glass petri dish (Pyrex) and scored under a dissection microscope (Nikon SMZ1500) for powder color and blood-feeding status. Females were scored as blood-fed if fresh blood was present in the abdomen. Percent biting was calculated as females freshly blood-fed/total females in each respective treatment group.

### Quantification and statistical analysis

All statistical analysis was performed using GraphPad Prism version 10. Data collected as percentage of total are shown as median with range. Data collected as raw values are shown as mean ± SEM or mean ± SD. Details of statistical methods are reported in the figure legends.

## Results

**Fig. 1:**
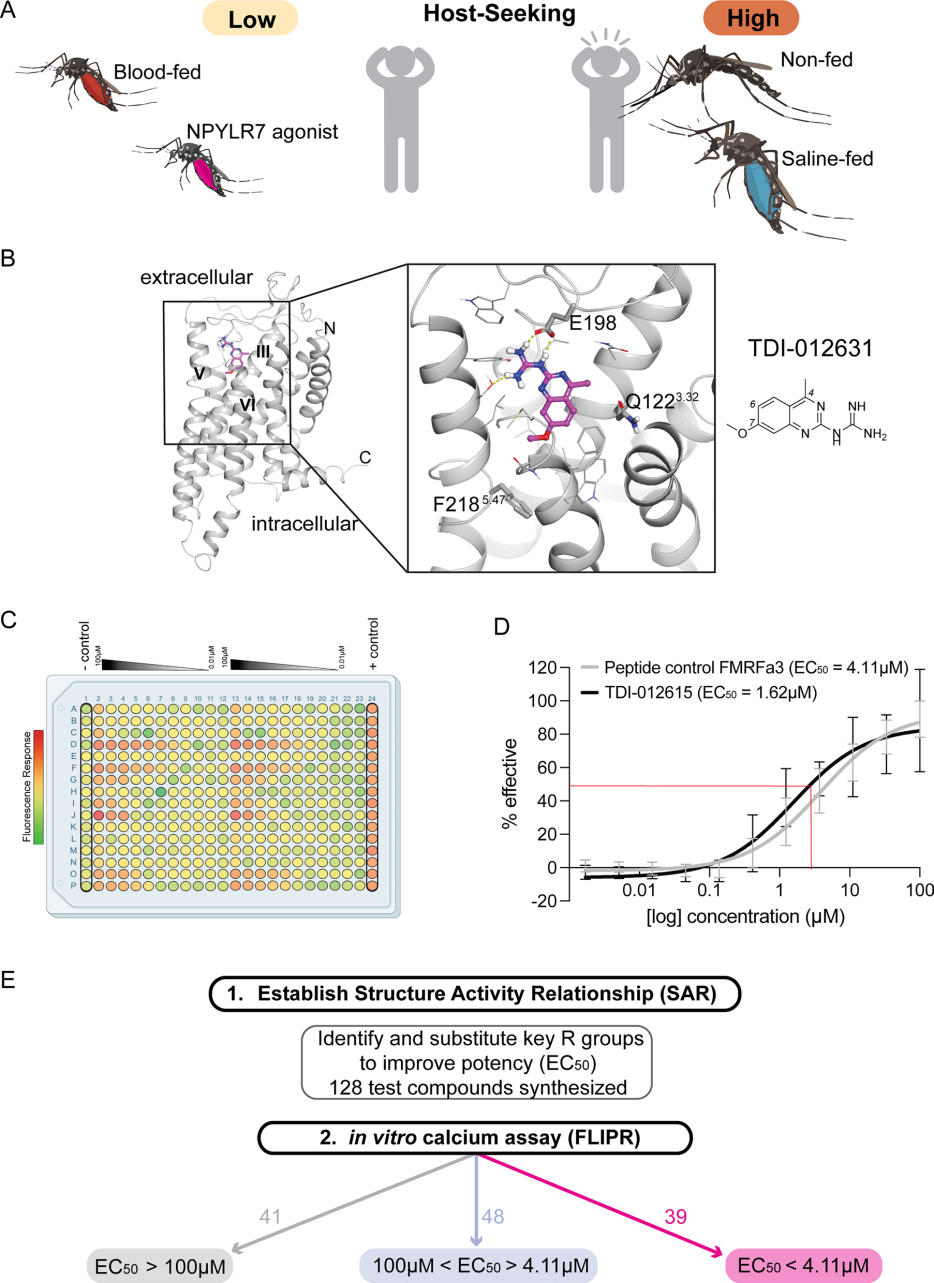
Structure-guided design to synthesize novel NPYLR7 agonists. **A** Representation of *Ae. aegypti* host-seeking behavior by feeding status. **B** NPYLR7-predicted structure model with compound TDI-012631 bound (left) and predicted side-chain interactions (right). **C** Representative 384-well plate layout from in vitro screen displaying raw fluorescence units (RFU) corresponding to test compounds ranging from concentration of 100 to 0 μM from left to right. Negative control (column 1) is measured as response to assay buffer alone, and positive control (column 24) as response to 10 μM dose of FMRFa3, an endogenous peptide activator of NPYLR7. **D** Semilogarithmic curves of compound TDI-012615 (EC_50_ = 1.62 μM, black line) and FMRFa3 (peptide control) sigmoidal curve (EC_50_ = 4.11 μM, gray line). **E** Outline of in vitro screening for 128 newly synthesized NPYLR7 agonists binned according to in vitro EC_50_

We previously showed that pharmacological activation of NPYLR7 inhibited *Ae. aegypti* mosquito host-seeking when delivered in a non-nutritive saline meal, and we identified small-molecule NPYLR7 agonists that inhibit host-seeking independent of nutrient consumption (Fig. 1A). However, the most potent of these compounds (TDI-012631) had an EC_50_ in activating NPYLR7 in vitro of 19.6 μM and was behaviorally active only when fed at high micromolar doses (> 30 μM). We therefore set out to design and evaluate analogs of this compound to identify those with improved potency. We generated a docking model of TDI-012631 bound to NPYLR7 to identify protein–ligand interactions that could inform analog design. The docking model showed that the quinazoline core occupies a hydrophobic pocket in the orthosteric binding site formed by transmembrane helices 3, 5, and 6. The guanidine substituent is stabilized by a salt bridge interaction with the Glu198 side chain in the EL2 loop, while the 4-position methyl group is pointed toward Gln122^3.32^ and 7-position methoxy group is oriented toward Phe218^5.47^ (Fig. 1B). This docking model is in agreement with early structure–activity relationship data for close analogs of TDI-012631, where the quinazoline core and guanidine appear to form a minimum pharmacophore. Indeed, replacement of the guanidine group with an amine (compound TDI-012610) causes a loss of potency. Newly synthesized analogs were then tested using a calcium-based HEK293T assay to determine their in vitro potency relative to FMRFa3, an endogenous peptide ligand of NPYLR7 (Fig. 1C and D). We used the original reference compound, TDI-012631, as a benchmark for potency. Based on the in vitro assay, compounds were grouped by EC_50_ value into those that showed low sensitivity or potency (> 100 μM), those with in vitro EC_50_ values < 100 μM but > 4.11 μM (TDI-012631, reference compound), and those with EC_50_ values < 4.11 μM.

**Fig. 2:**
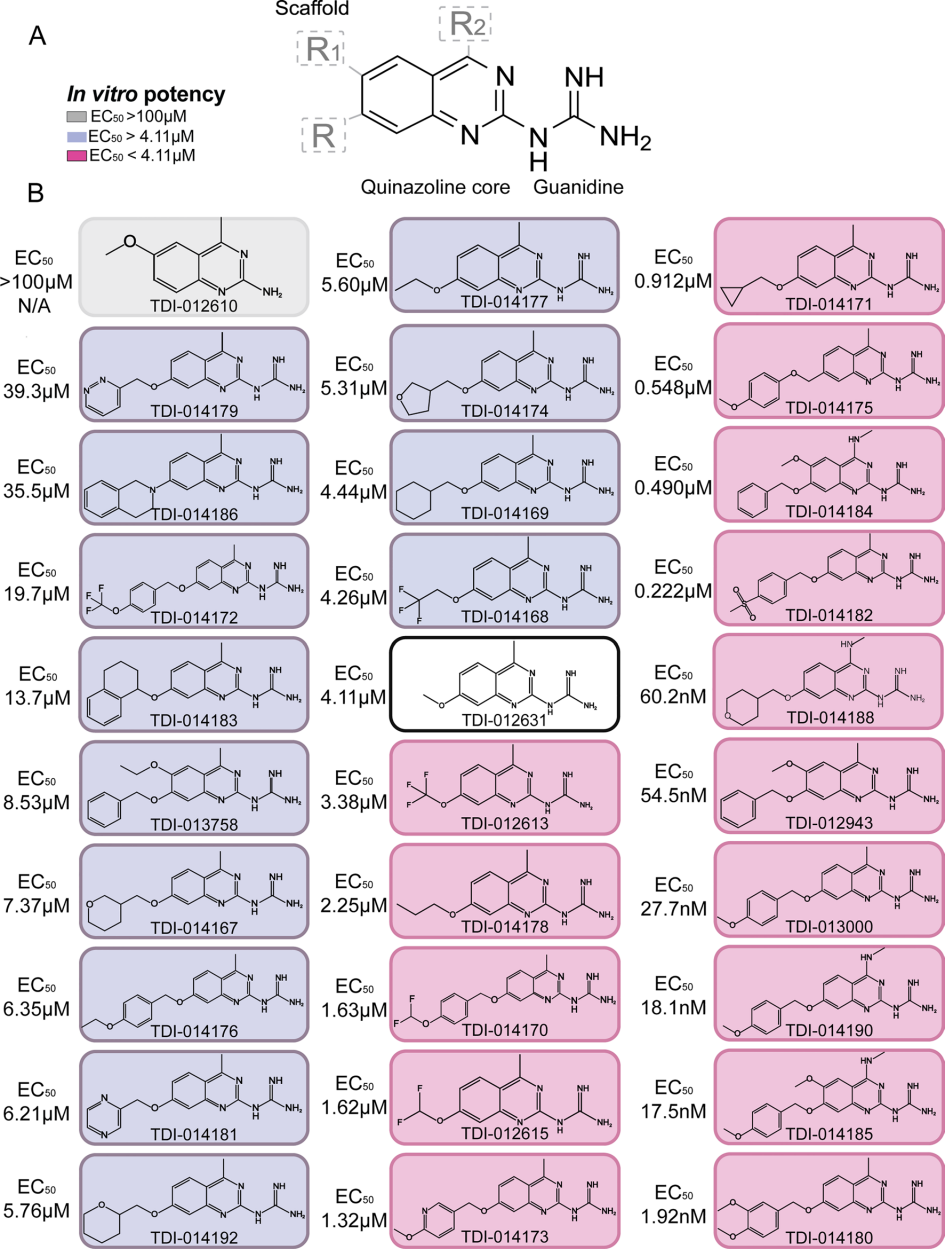
Small-molecule NPYLR7 agonists tested in vivo. **A** Lead compound scaffold with conserved quinazoline core, guanidine group, and R groups tested for substitutions. Shading in legend indicates EC_50_ in **B**. **B** Chemical structures with corresponding in vitro EC_50_ of each compound tested in behavioral assays arranged with the highest value at the top left to the lowest value at the bottom right. TDI-012631 (center, black border) is the initial reference compound

Analogs of TDI-012631 were designed by maintaining the quinazoline core and the guanidine substituent that formed critical interactions in the docking model and modifying the substituents around the core at the 4 (R2), 6 (R1), and 7 (R) positions (Fig. 2A). Bulkier groups were explored for R to extend into the deeper pocket, while the hydrogen-bond-donating NHCH_3_ group was introduced for R2 to interact with the Gln122^3.32^ side chain (Fig. 2A). Over 100 compounds were designed and synthesized. To identify compounds with in vivo activity, 30 compounds were selected for testing in host-seeking assays. This group included compounds with predicted EC_50_ values ranging from 39.3 μM to 1.92 nM as well as a negative control compound from the > 100 μM group (Fig. 2B).

**Fig. 3:**
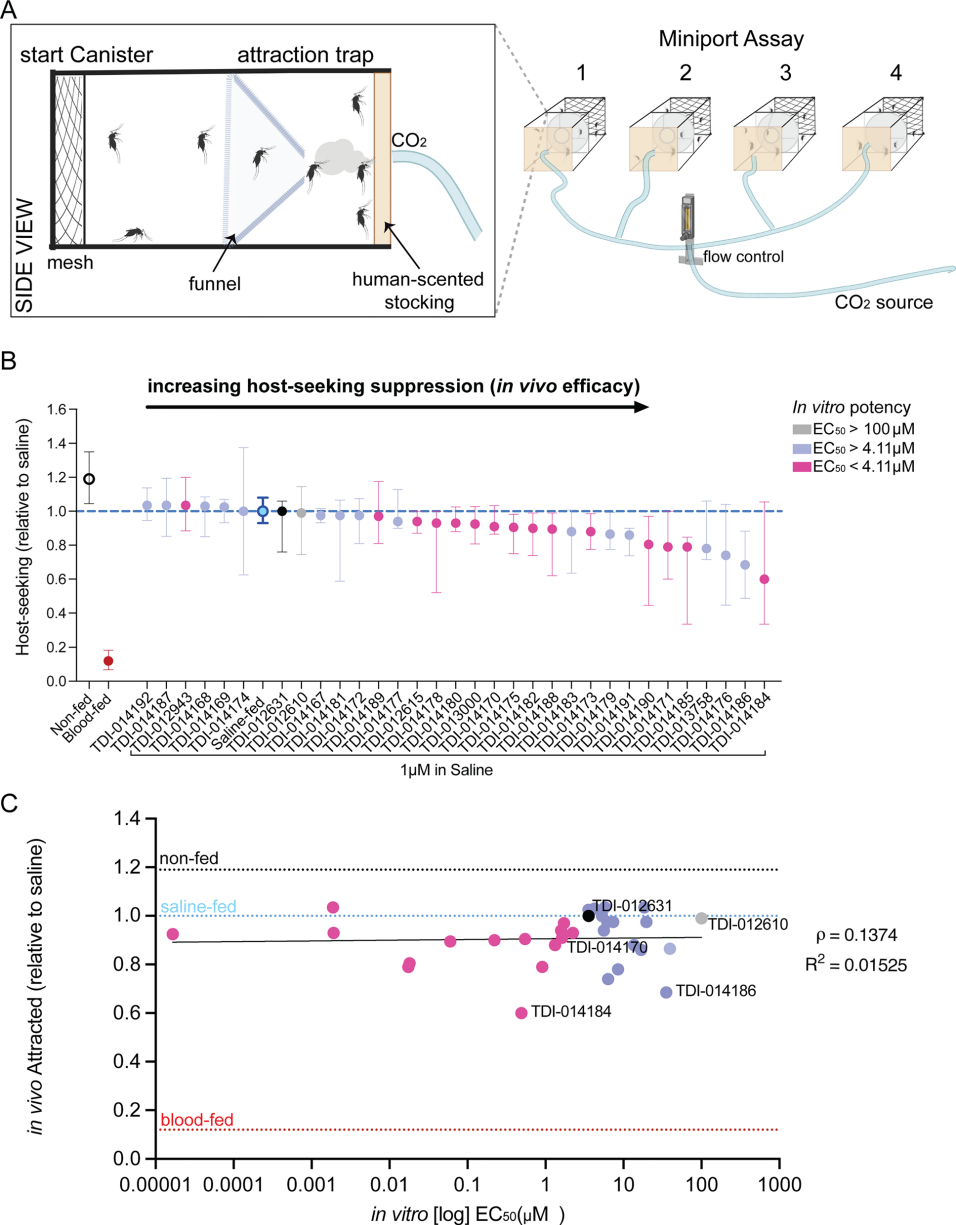
Miniport olfactometer screening assay to identify compounds that reduce attraction to human host cues. **A** Schematic of the miniport olfactometer host-seeking assay. Inset depicts start canister and attraction trap modular components for each experiment. Mosquitoes not drawn to scale. **B** Host-seeking relative to saline (test compound % host-seeking/average matched saline % host-seeking). (Median with interquartile range, *n* = 4–65 replicates, 15–20 females/replicate) **C** Correlation of in vitro potency to in vivo host-seeking in *Ae. aegypti*. Spearman correlation coefficient (ρ) = 0.1374, *P* > 0.05, slope = 0.002942, *R*^2^ = 0.01525

To prioritize candidates with the highest levels of in vivo efficacy, we performed a screening miniport olfactometer assay that allowed us to test mosquito host-seeking behavior in four conditions in parallel (Fig. 3A). In this assay we provided two host cues, CO_2_ and human odor collected on a worn nylon stocking. Animals were scored as attracted if they flew from the starting canister into the attraction trap next to the source of the host cues. Animals were fed each compound at a dose of 1 μM in non-nutritive saline 2 days prior to testing, then scored and weighed to ensure that compounds did not affect meal palatability or consumption (Supplemental Figure 1). Animals were allowed to recover for 2 days before host-seeking assays were performed, to ensure that suppression was not attributable to abdominal distension from meal consumption. Using the miniport olfactometer assay, we identified compounds that inhibit host-seeking relative to saline alone (Fig. 3B). Consistent with previous work, the original lead compound TDI-012631 was not active at a dose of 1 μM. We plotted the relationship between in vitro EC_50_ and in vivo efficacy in the miniport assay and found that, although we identified compounds that were more effective than TDI-012631, there was no predictive relationship between in vitro EC_50_ and in vivo efficacy (Fig. 3C).

**Fig. 4:**
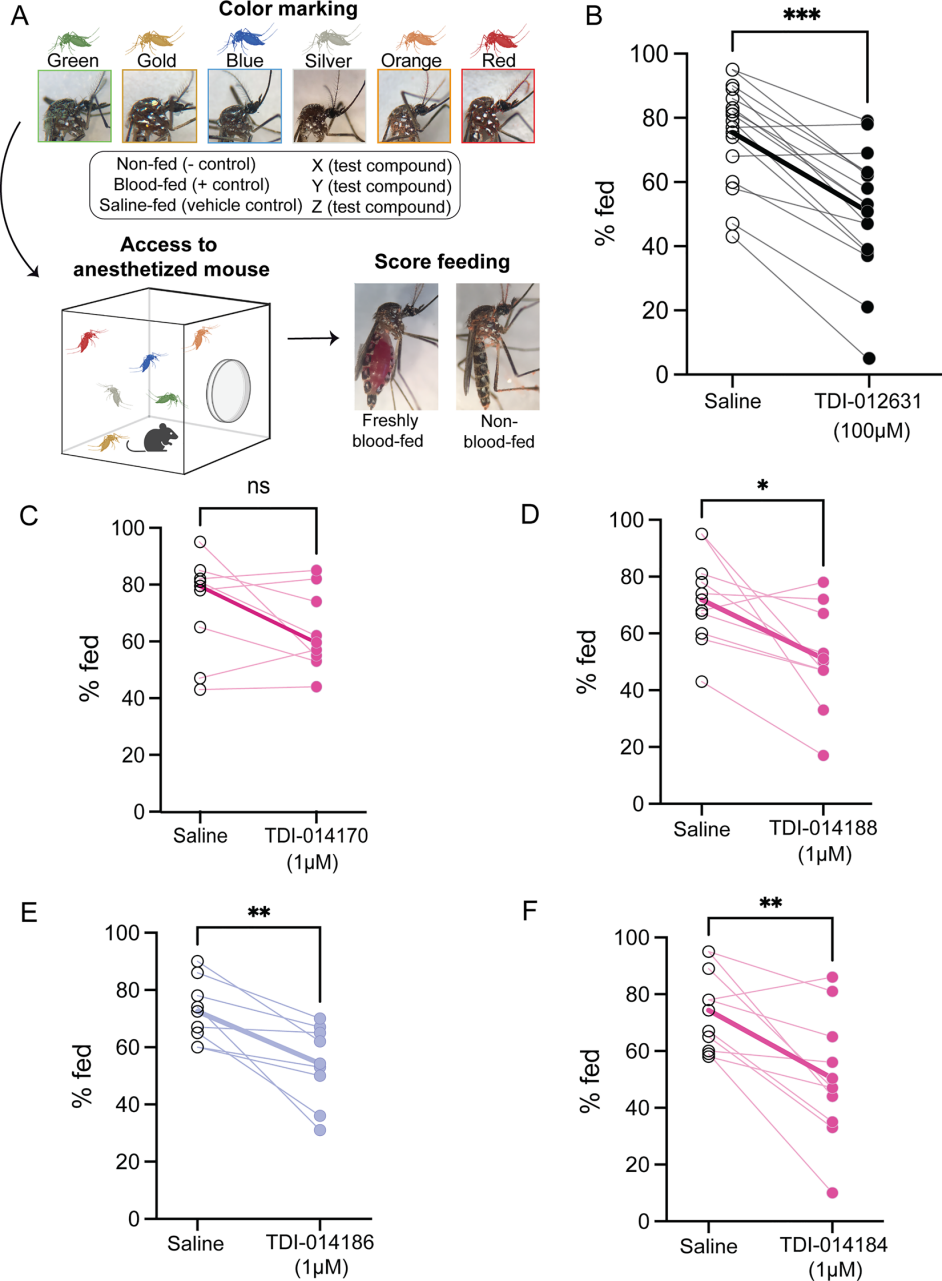
Novel NPYLR7 agonists reduce blood-feeding from a live host when fed at a dose of 1 μM. **A** Schematic of “mouse-in-cage” biting assay workflow depicting powder color assignment, exposure to mouse, and scoring of fresh blood in the abdomen. **B**–**F** Percentage of females that freshly blood-fed on an anesthetized mouse after 15 min exposure. Females were fed the indicated meal 48 h prior to the experiment. **C, D, F** High-potency and (**E**) medium-potency in vitro compounds (see Fig. 2B). Bold lines represent saline group mean versus test group mean. Total of 15–20 females per replicate. Mann–Whitney test: **B** *n* = 13 replicates, ***P = 0.0003; **C** *n* = 9 replicates; ns, not significant; *P* = 0.2868, **D** *n* = 11 replicates, *P = 0.0120. **E** *n* = 9 replicates, **P = 0.0097, **F** *n* = 11 replicates; **P = 0.0055

We next asked whether compounds that suppressed attraction to host cues in the miniport olfactometer assay could also block blood-feeding from a live host using a mouse-in-cage assay in which mosquitoes are fed test compounds and presented with an opportunity to blood-feed from an anesthetized mouse 2 days later. Non-blood-fed females were robustly attracted to the mouse and blood-fed at high rates (91.0 ± 1.92%), while females that were naturally suppressed after a meal of sheep blood rarely fed (7.0 ± 1.21%) (Data S1). At the end of each experiment, mosquitoes were collected and scored for the presence of fresh blood in their abdomen, indicating that they successfully fed on the blood of the mouse (Fig. 4A). We replicated the finding that TDI-012631 inhibited blood-feeding when fed to mosquitoes at a dose of 100 μM (Fig. 4B) [2]. Although TDI-014170 showed high in vitro potency (EC_50_ = 1.63 μM), this compound did not reduce host-seeking in our miniport olfactometer screening assay nor did it significantly reduce biting in the mouse-in-cage assay when fed at a dose of 1 μM (Fig. 4C). However, we identified three novel small-molecule NPYLR7 agonists that reduced biting behavior when fed at a 1 μM dose (Fig. 4D–F). These included TDI-014188 and TDI-014186, which were the two compounds that showed the largest effect in the miniport host-seeking assay (Fig. 3B). Two of the three active compounds, TDI-014184 (EC_50_ = 0.490 μM) (Fig. 4F) and TDI-014188 (EC_50_ = 60.2 nM) (Fig. 4D), showed significantly improved in vitro potency relative to the reference compound. Surprisingly, TDI-014186 (Fig. 4E) effectively reduced biting despite showing lower in vitro potency (EC_50_ = 35.5 μM) relative to the reference compound.

## Discussion

Multiple mosquito genera contribute to the spread of human disease. *Aedes* mosquitoes can transmit the dangerous arboviruses yellow fever, dengue, Zika, and chikungunya [21, 22]. The emergence and geographical spread of these viruses are critical concerns for global public health [21, 23, 24]. Although much research has been dedicated to developing vaccines and prophylactic or therapeutic drugs to treat and prevent these diseases there are currently no drugs to treat the viruses spread by *Ae. aegypti.* Although there is an effective vaccine against the yellow fever virus and there have been significant improvements in recent dengue vaccination strategies [25], preventing mosquito bites remains essential for reducing disease transmission. Current strategies to control mosquito populations rely on toxic pesticides that decline in efficacy as mosquito populations rapidly develop resistance [26]. More recently developed approaches involve the release of mosquitoes rendered either sterile or unable to transmit pathogens [27, 28] or releasing transgenic animals with altered immune or reproductive function [29–31]. However, ethical, environmental, and regulatory concerns remain issues in the deployment of such transgenic mosquitoes. Although each of these approaches has shown some success, there remains a major unmet need to develop innovative and complementary strategies for integrated mosquito control.

The most prevalent chemical control methods include synthetic insecticides or repellents, and previous studies have focused primarily on determining the pharmacokinetic properties of these compounds in non-target vertebrates at doses lethal to the target insect [32, 33]. Lethality contributes to the evolution of resistance by selecting for individuals carrying resistance alleles that can escape lethality [34, 35]. Most insecticides are limited to a few chemical classes with similar mechanisms of action and the World Health Organization has urged the development of new mosquito control techniques that exploit novel chemical classes [36]. Increasingly, new strategies have focused on non-lethal methods of pest control [37, 38]. This category includes the small-molecule NPYLR7 agonists tested in this study, which reduce the drive to bite without killing the mosquito. However, there is a need for relevant drug discovery assays in insects to characterize the utility and potential for development of non-lethal chemicals. In traditional drug discovery and development, in vitro assays to determine potency are normally followed by assays to determine target engagement in cells and pharmacokinetic/pharmacodynamic (PK/PD) properties to evaluate how compounds are absorbed, metabolized, and eliminated from specific tissues in the body. There are examples of insect models for drug discovery; silkworms (*Bombyx mori*) have been used as a model for drug toxicity and show responses to hepatotoxic drugs that are consistent with mammalian models [39, 40]. More recent work has established novel models for pharmacokinetic assays in mosquitoes by delivering ivermectin and cytochrome P450 modulators in blood, using liquid chromatography with tandem mass spectrometry (LC–MS/MS) to quantify clearance rates after feeding and modeling primary pharmacokinetic parameters and drug/drug interactions. Ivermectin clearance kinetics differ between mosquitoes and mammals, and individual P450 modulators were eliminated with differing kinetics in mosquitoes [41]. However, detection remains a limiting factor. Drug concentrations are higher than those that can be obtained in the blood of humans receiving a regular dose of ivermectin and because whole mosquito specimens were required for analysis it is not yet possible to achieve tissue-specific resolution of drug occupancy/clearance. We found that in vitro EC_50_ was not predictive of behavioral effect among the compounds tested in our assays and future work to characterize the clearance rates of the compounds described here may clarify the relationship between EC_50_ in our cell-based assay and behavioral effect. Compounds like TDI-014170 with high in vitro potency may be inactive in vivo because they lack bioavailability in the mosquito, or have a half-life that is too short to be captured when assays are performed 2 days after feeding. Compounds like TDI-014186 that reduced host-seeking behavior despite modest in vitro EC_50_ may achieve in vivo potency through sequestration in specific target tissues.

NPY-like receptors are present in many insects including other blood-feeding arthropods [42, 43]. This suggests that NPY pathways may represent a conserved biological mechanism that could be targeted for the development of a more generalized strategy to suppress attraction to humans across multiple species of mosquitoes and ticks. To ensure that beneficial insects are not impacted, in vitro assays to identify compounds that show minimal cross-activation of related receptors in other insect species will be crucial. Delivering these compounds via human odor-baited traps will also ensure that beneficial insects are not targeted; a method is already in use in attractive toxic sugar baits [44–47]. These strategies rely on toxic pesticides that decline in efficacy as mosquito populations rapidly develop resistance [26]. NPYLR7 agonists could be delivered in attractive *non*toxic sugar baits to inhibit mosquito attraction to human hosts and reduce the number of mosquitoes actively biting. Additionally, mosquitoes that ingest our compounds in a protein-free formulation do not produce eggs, thereby reducing the overall mosquito population size. Previous mathematical modeling has shown that both reducing the number of bites and reducing the effective mosquito population size have a dramatic effect on disease transmission [48].

## Conclusions

By combining advanced molecular modeling, medicinal chemistry, and precise deployment strategies, there is potential to develop targeted and environmentally responsible solutions for managing mosquito populations. Continued research into the structural aspects of these compounds and their receptor interactions will pave the way for the development of next-generation insect control agents with broader applicability and improved efficacy. Further study of the structural relationship between these compounds and *Ae. aegypti* NPYLR7 may help to identify new highly potent compounds and the rules by which they activate their cognate receptors.

### Supplementary Information

Supplementary Material 1: Figure 1. Small-molecule NPYLR7 agonists do not affect meal palatability. (**A**) Percent of females feeding to repletion and (B) weight per female after feeding on the indicated meal used for testing in Miniport olfactometer experiments in Figure 3B. (C) Percent of females feeding to repletion and (D) weight per female after feeding on the indicated meal used for testing in live host assays in Figure 4. Data are shown as mean with standard deviation. *n* = 2–22 replicate cages, 60–150 females/cage. Females were weighed in groups of five to ensure reliable readings. Kruskal–Wallis with Dunn’s multiple comparisons to saline-fed group, ns *P* > 0.01, * *P* < 0.01, *** *P* < 0.0001. (PNG 321 KB)

Supplementary Material 2.

Supplementary Material 3.

## Data Availability

All data generated or analyzed during this study are included in this published article and its supplementary information files. Raw data are provided in Data S1. Cartoons in Figs. 1, 3, and 4 were created with BioRender.com. Chemical structures in Figs. 1 and 2 were generated with ChemDraw (version 21.0.0). The Schrödinger Suite was used for protein and ligand preparations, docking, MD, and FEP+ calculations (release 2020–4, Maestro, Schrödinger LLC, New York, NY).
