# Supplementary figures and images for: Next-generation neuropeptide Y receptor small-molecule agonists inhibit mosquito-biting behavior
Source: Parasit Vectors. 2024 Jun 28;17:276. doi: 10.1186/s13071-024-06347-w (PMC11212260; doi:10.1186/s13071-024-06347-w)

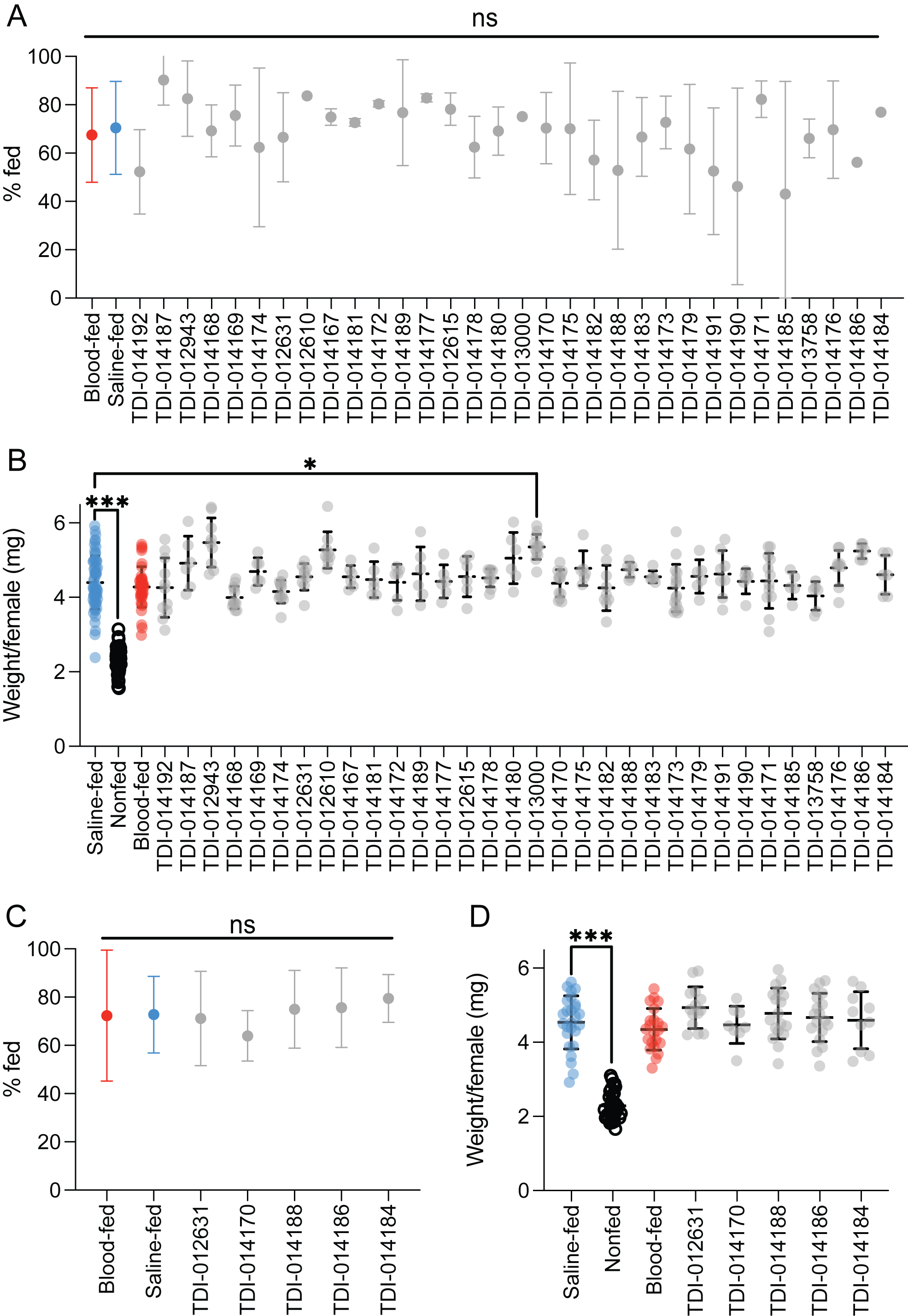

Supplement: Supplementary file 1 — Supplementary Material 1: Figure 1. Small-molecule NPYLR7 agonists do not affect meal palatability. (A) Percent of females feeding to repletion and (B) weight per female after feeding on the indicated meal used for testing in Miniport olfactometer experiments in Figure 3B. (C) Percent of females feeding to repletion and (D) weight per female after feeding on the indicated meal used for testing in live host assays in Figure 4. Data are shown as mean with standard deviation. n = 2–22 replicate cages, 60–150 females/cage. Females were weighed in groups of five to ensure reliable readings. Kruskal–Wallis with Dunn’s multiple comparisons to saline-fed group, ns P > 0.01, * P < 0.01, *** P < 0.0001. (PNG 321 KB) [file 13071_2024_6347_MOESM1_ESM.png]
